# Revolutionizing transdermal drug delivery evaluation: the evolution from traditional models to skin organoids and the integration of artificial intelligence

**DOI:** 10.3389/fphar.2026.1855808

**Published:** 2026-09-28

**Authors:** Xinqiang Mo, Mingshi Qiu, Ru Song, Zhenjie Wu, Chunyan Liu, Linqi Su, Haiyi Wang, Rui Xue, Lu Yu, Maoyao Wang, Ziyang Zhu, Peng Wu, Jian Liu, Yibing Wang, Siyuan Yin

**Affiliations:** 1 Department of Plastic Surgery, The First Affiliated Hospital of Shandong First Medical University & Shandong Provincial Qianfoshan Hospital, Jinan, Shandong, P. R. China; 2 Jinan Clinical Research Center for Tissue Engineering Skin Regeneration and Wound Repair, Jinan, Shandong, P. R. China; 3 Department of Medical Dataology, School of Public Health, Cheeloo College of Medicine, Shandong University, Jinan, Shandong, P. R. China; 4 National Institute of Health and Medical Big Data, Jinan, Shandong, P. R. China; 5 First Clinical Medical College, Shandong University of Traditional Chinese Medicine, Jinan, China; 6 Department of Stomatology, Shandong Provincial Hospital Affiliated to Shandong First Medical University, Jinan, China

**Keywords:** artificial intelligence, Franz diffusion cell, *in vitro* permeation test, skin organoids, transdermal drug delivery

## Abstract

This review examines the evolution of evaluation systems for transdermal drug delivery systems (TDDS) alongside recent advances in skin organoids (SkOs). We discuss the potential of SkOs as *in vitro* models, with particular attention to their structural complexity and their ability to recapitulate appendage-associated transport pathways more effectively than traditional animal models and reconstructed human epidermis (RHE). We also summarize current fabrication strategies, physiological validation, and the main translational limitations of SkOs. In addition, we discuss how artificial intelligence (AI), computer vision, and digital twin technologies may help address current challenges in standardization and quality control. Self-assembling SkOs derived from induced pluripotent stem cells represent a promising candidate advance for future TDDS evaluation. Although limitations in maturation remain, the integration of SkOs with AI may support the development of a more standardized, high-throughput, and predictive platform for transdermal drug delivery research.

## 1 Introduction

Transdermal drug delivery refers to the administration of drugs onto the skin surface so that they pass through the skin barrier, especially the stratum corneum, into deeper skin tissues or the systemic circulation to exert local or systemic therapeutic effects (Prausnitz and Langer, 2008; Pastore et al., 2015). Within the TDDS evaluation framework, *in vitro* permeation and release studies play a central role in predicting clinical performance (Franz, 1975). Despite the promise of transdermal delivery, current clinical applications remain largely limited to drugs that are highly lipophilic, low in molecular weight, and effective at low doses. Expanding this approach to macromolecules and hydrophilic compounds depends not only on advances in penetration-enhancing strategies and delivery systems, but also on the availability of evaluation models with greater physiological relevance (Phatale et al., 2022; Lane, 2024).

Within the current evaluation paradigm, *in vitro* permeation devices—particularly the Franz diffusion cell—are already well established. By contrast, the skin models used in these systems still fall short of meeting increasingly complex research and development needs. This gap has created a strong demand for more physiologically relevant alternatives. In practice, existing *ex vivo* skin models, including human and porcine skin, face several limitations. Restricted tissue availability and substantial inter-individual variability reduce experimental efficiency, while the gradual loss of tissue viability compromises predictive performance (Simon and Maibach, 2000; Jung and Maibach, 2015). As a result, there is a clear need for improved surrogate skin models. Ideally, such models should reproduce not only the biomechanical properties of native skin, such as elasticity and toughness, which are important for evaluating delivery technologies such as microneedles, but also key physiological structures and biochemical functions. Better replication of these features would improve in vitro–in vivo correlation (IVIVC) and increase the clinical relevance of preclinical evaluation. At present, however, existing models remain insufficient for the assessment of many complex transdermal systems. A generalized workflow of TDDS evaluation, spanning physicochemical characterization, *in vitro* release and skin permeation testing, *in vivo* pharmacokinetic/pharmacodynamic assessment, and safety evaluation, is summarized in Figure 1.

**FIGURE 1:**
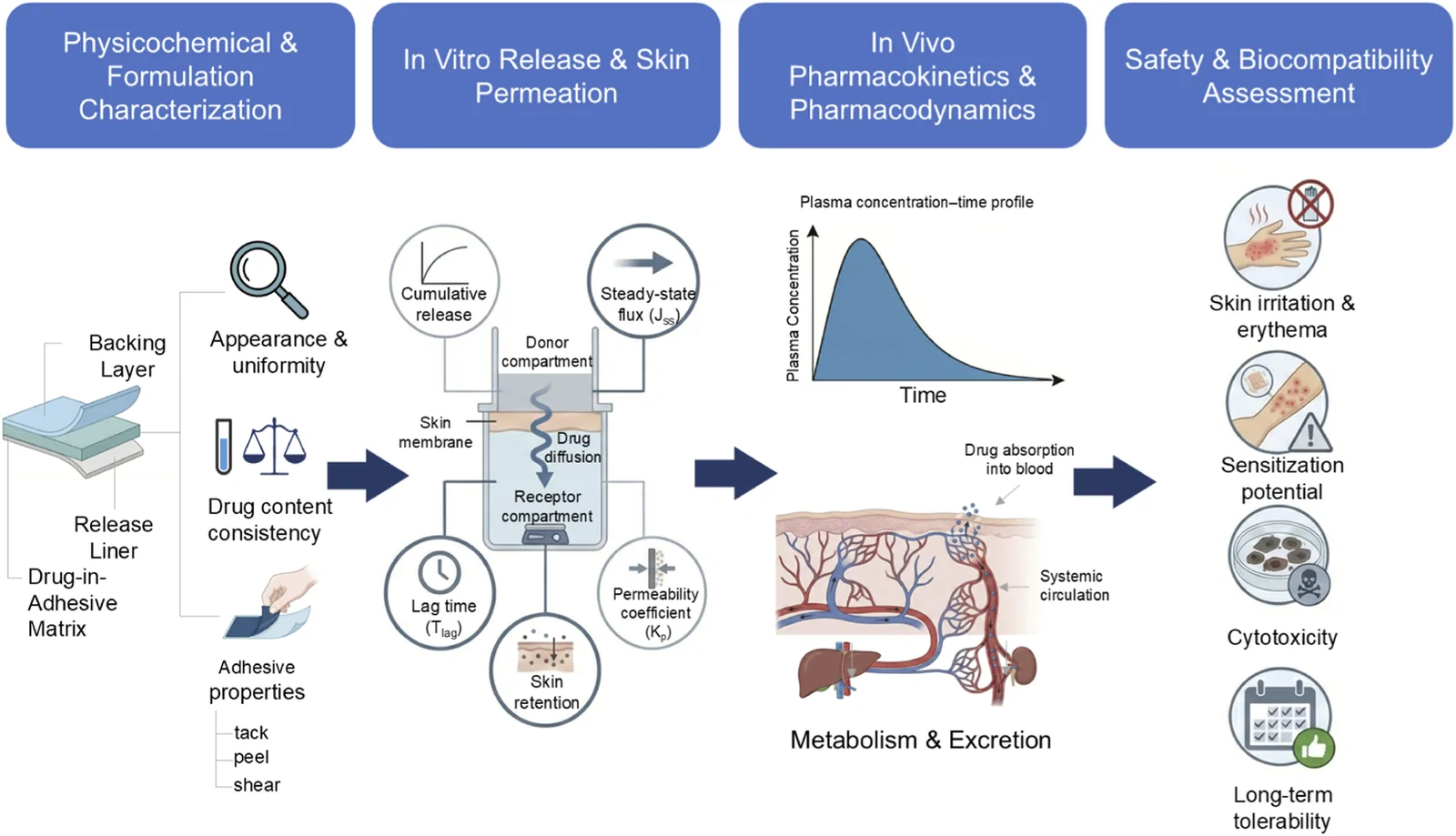
Overview of the TDDS evaluation framework. Schematic overview of the major components involved in transdermal drug delivery system (TDDS) evaluation, including physicochemical and formulation characterization, *in vitro* release and skin permeation testing, *in vivo* pharmacokinetic/pharmacodynamic assessment, and safety/biocompatibility evaluation. Representative endpoints include appearance and uniformity, drug content consistency, adhesive performance, cumulative release, steady-state flux, lag time, permeability coefficient, skin retention, systemic exposure, and local or systemic safety outcomes. This framework highlights the central position of *in vitro* skin permeation testing within the broader preclinical evaluation of TDDS.

Although reconstructed human epidermis (RHE) models still have limitations in drug permeation studies, they have successfully replaced traditional animal testing in skin corrosion and irritation assays (OECD, 2025b) and are now widely used in the cosmetics field. This progress highlights the scientific and commercial value of engineered skin models. The emergence of induced pluripotent stem cell (iPSC)-derived skin organoids (SkOs) in 2020 created a new opportunity to address long-standing limitations in TDDS evaluation (Lee et al., 2020). Through self-organization, SkOs can generate complex structures, including hair follicles, sebaceous glands, and sensory neurons. Compared with RHE models, they provide greater anatomical depth and physiological complexity, which may improve the evaluation of transdermal drug delivery (Shafiee et al., 2024).

To define the scope of this narrative review, we conducted a targeted literature search rather than a formal systematic review. Relevant studies were identified from PubMed, Web of Science, Scopus, and Google Scholar, covering publications from the development of conventional *in vitro* skin models to June 2026. Search terms included combinations of “skin organoid”, “skin organoids”, “SkOs”, “induced pluripotent stem cell”, “iPSC-derived skin”, “reconstructed human epidermis”, “full-thickness skin model”, “transdermal drug delivery”, “*in vitro* permeation testing”, “Franz diffusion cell”, “skin-on-a-chip”, “PBPK”, “digital twin”, “artificial intelligence”, “machine learning”, and “computer vision”. We prioritized original studies, protocols, regulatory guidance, and review articles that were directly relevant to skin organoid construction, skin disease modeling, barrier function, TDDS evaluation, IVPT methodology, model standardization, and digitally enabled quality control. Additional references were identified by manually screening the reference lists of key articles. Because the purpose of this article is to provide a narrative and forward-looking synthesis rather than a quantitative systematic review or meta-analysis, studies were selected according to their relevance to the conceptual framework of SkOs as emerging human-relevant platforms for TDDS research.

In this review, we summarize the evolution and current landscape of TDDS evaluation models and skin organoids, with a particular focus on their potential applications in complex drug delivery settings. We also discuss key challenges that still limit the broader use of SkOs, including high production costs and limited standardization. In addition, we examine how artificial intelligence (AI) may contribute to organoid quality control and permeation prediction. Together, these advances may support the development of skin organoids into more robust and scalable platforms for TDDS evaluation. To provide an overall historical framework for the discussion that follows, the major milestones in TDDS evaluation, *in vitro* skin model development, regulatory change, and digitally enabled translational concepts are summarized in Figure 2.

**FIGURE 2:**
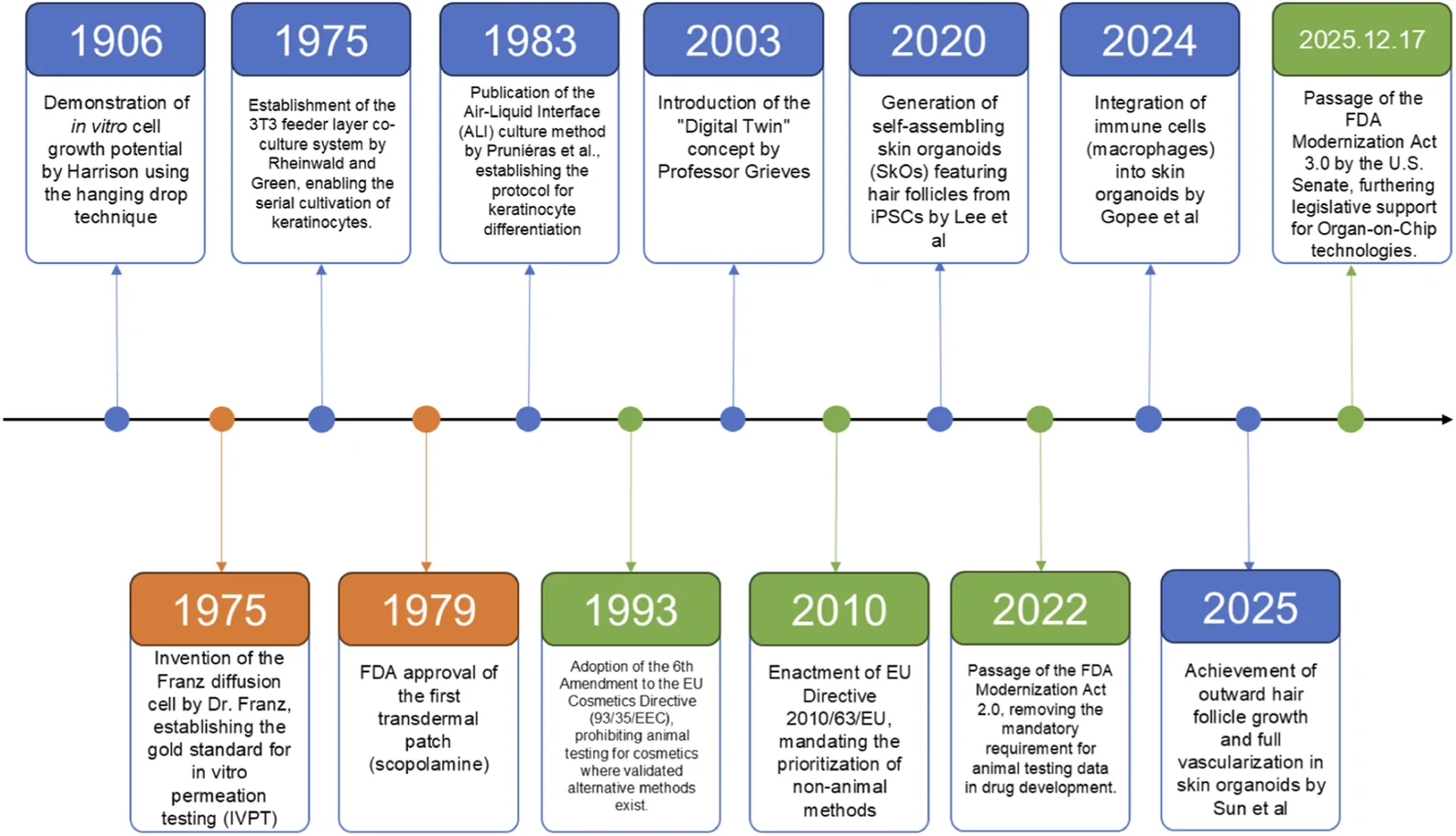
Major milestones in the evolution of TDDS evaluation, *in vitro* skin model development, regulatory change, and digitally enabled translational concepts. Timeline summarizing representative milestones that shaped the field, spanning early *in vitro* cell culture methods, keratinocyte feeder-layer systems, air-liquid interface culture, and the Franz diffusion cell, as well as the approval of the first transdermal patch, the emergence of skin organoids, the introduction of the digital twin concept, and recent legislative shifts supporting non-animal testing strategies. Together, these milestones illustrate the progressive convergence of biological modeling, engineering innovation, regulatory reform, and digital intelligence in next-generation TDDS evaluation.

## 2 Evolution of transdermal drug delivery evaluation systems

### 2.1 Historical stages of TDDS evaluation

The development of TDDS evaluation reflects the co-evolution of methodological standardization and skin-model innovation. Early studies relied mainly on exploratory *in vivo* skin absorption experiments and relatively fragmented *in vitro* diffusion methods, resulting in limited data comparability and weak predictive value for human skin permeation. With the progressive establishment of the Franz diffusion cell, *in vitro* permeation testing (IVPT), pharmacopeial performance tests, and product-specific bioequivalence guidance for topical products, TDDS evaluation has gradually evolved into a more quantitative, reproducible, and regulatory-relevant experimental framework. In this process, the focus has expanded from optimizing diffusion devices and experimental procedures to selecting biological models that more faithfully reproduce the human skin barrier. In other words, the evolution of TDDS evaluation is not only a technical advance in measurement, but also a continuous renewal of skin models in terms of human relevance, barrier function, and standardizability.

#### 2.1.1 Phase I: the exploration period (pre-1975)

Before 1975, transdermal drug delivery research was still at an early exploratory stage, and studies of skin absorption lacked unified methodological standards. Many investigations from this period relied on relatively crude *in vivo* approaches, such as measuring urinary drug excretion, or on *in vitro* immersion assays performed under non-physiological conditions. As a result, these methods often failed to distinguish superficial drug adsorption from true transmembrane permeation. This period was therefore marked by methodological fragmentation, inconsistent data, and a strong dependence on animal models. Researchers explored a wide range of systems, from animal skin to early synthetic membranes, but both the experimental devices and the associated protocols remained underdeveloped (Prausnitz and Langer, 2008; Wiedersberg and Guy, 2014). Although early investigators such as Tregear et al. attempted to establish physical models of skin permeability, these efforts were limited by the available analytical tools and did not lead to broadly adopted standards.

#### 2.1.2 Phase II: establishment of technologies and standards (1975–pre-2020s)

The year 1975 marked an important turning point in TDDS evaluation. In that year, Franz systematically discussed the relevance of *in vitro* percutaneous absorption data to human skin and promoted the use of diffusion-cell methods in skin absorption research, allowing drug permeation across the skin to be quantitatively evaluated as a measurable physicochemical process (Franz, 1975). This work laid an important experimental foundation for modern *in vitro* permeation testing (IVPT). With the subsequent development of standardized skin absorption guidelines, pharmacopeial performance tests, and bioequivalence guidance for topical formulations, IVPT evolved from a permeation assay used primarily in basic research into an important methodological platform for evaluating the performance of topical formulations and transdermal drug delivery systems.

After the Franz diffusion cell established the experimental basis for *in vitro* percutaneous absorption research, the methodological role of IVPT gradually expanded from basic permeation measurement to performance evaluation and equivalence assessment of topical products. In 2004, the OECD issued TG 428, Skin Absorption: *In Vitro* Method, which systematically specified key elements of *in vitro* skin absorption studies, including skin source, sample preparation, barrier integrity, receptor fluid, temperature control, dosing conditions, and mass balance. This guideline moved *in vitro* skin permeation studies from relatively fragmented laboratory practices into a more comparable and reproducible standard framework (OECD, 2004). Within the subsequently developed pharmacopeial framework, USP <1724>, Semisolid Drug Products-Performance Tests, incorporated *in vitro* performance testing of semisolid products into the USP-NF system, further linking IVPT with topical product quality, product performance, and batch consistency (United States Pharmacopeia, 2023). In the stage of topical-product bioequivalence assessment, the FDA product-specific guidance for acyclovir topical cream first recommended IVPT in 2014 and later clarified that appropriately validated IVPT studies can be used to compare the rate and extent of drug permeation from test and reference products across *ex vivo* human skin (U.S. Food and Drug Administration, 2022b). In 2022, the FDA issued draft guidance on IVPT studies for topical drug products submitted in ANDAs, further discussing IVPT method development, validation, conduct, data analysis, and reporting requirements (U.S. Food and Drug Administration, 2022a). The EMA guideline on quality and equivalence of topical products, adopted in 2024 and effective from 2 April 2025, further refined requirements for donor number, replicate design, skin integrity, surface temperature, receptor medium, and statistical acceptance criteria in IVPT (European Medicines Agency, 2024). Thus, the evolution of IVPT has not merely involved improvements in testing devices, but rather a progressive strengthening of standardization, quality control, and regulatory interpretability. As this methodological framework matures, the key determinant of predictive performance increasingly shifts toward whether the skin model itself can faithfully represent the human skin barrier.

As IVPT has become increasingly standardized, the importance of the skin model itself has become more apparent. Early rodent skin models often overestimated drug permeation because of their thin stratum corneum and high hair follicle density, which can generate pronounced shunt effects (Bartek et al., 1972; Jung and Maibach, 2015). Porcine skin has been widely used because it is morphologically closer to human skin, but differences in stratum corneum lipid organization, sweat gland structure, and appendage distribution still limit its extrapolation to complex formulations, microneedles, and nanocarrier systems (Simon and Maibach, 2000; Bouwstra et al., 1991).

By contrast, *ex vivo* human skin has long been regarded as the most relevant reference material for IVPT, particularly for evaluating the permeation behavior of topical formulations and TDDS across a real human skin barrier (Franz et al., 2009; Raney et al., 2015). However, *ex vivo* human skin is constrained by limited availability, ethical requirements, donor age, anatomical site, storage conditions, and loss of long-term viability. Modern IVPT therefore increasingly emphasizes control of donor variability, paired adjacent skin sections from the same donor, pre-study integrity testing, and predefined data-exclusion criteria. In this sense, contemporary IVPT is no longer a simple combination of a diffusion cell and a skin membrane, but a standardized evidentiary system composed of the skin model, experimental design, analytical method, and statistical evaluation.

To reduce biological variability, de-biologized synthetic membranes such as Strat-M™ have been used as early-stage formulation screening tools (Uchida et al., 2015). These membranes are useful for high-throughput and preliminary formulation screening, but they lack dynamic stratum corneum lipid organization, enzymatic activity, cellular responses, and the microenvironment of living skin. They are therefore difficult to use as core models for equivalence assessment (Haq et al., 2018; Arce et al., 2020). Meanwhile, three-dimensional RHE models constructed from human cells have been introduced into transdermal permeation and topical formulation research. RHE has achieved substantial success in skin toxicology and cosmetic testing and has also been explored for *in vitro* bioequivalence testing (Schreiber et al., 2005; Schäfer-Korting et al., 2006; Agonia et al., 2022). However, conventional RHE models generally lack a complete dermis, hair follicles, sebaceous glands, vasculature, and immune components, and their stratum corneum lipid composition and barrier strength still do not fully reproduce adult native skin. These models are therefore better suited for screening and complementary evaluation than as complete replacements for *ex vivo* human skin in standard IVPT. A comparison of RHE, full-thickness skin models, ex vivo human skin, and SkOs for TDDS evaluation is provided in Table 1.

**TABLE 1:** Comparison of RHE, full-thickness skin models, *ex vivo* human skin, and SkOs for TDDS evaluation.

Evaluation dimension	Reconstructed human epidermis (RHE)	Full-thickness skin models (FT skin models)	*Ex vivo* human skin	Skin organoids (SkOs)
Tissue architecture	Stratified epidermal structure, including basal, spinous, granular, and cornified layers	Epidermis plus dermal-like compartment, usually containing fibroblasts and collagen matrix	Native full-thickness skin structure, retaining epidermis, dermis, and part of subcutaneous tissue	Multilineage three-dimensional structure capable of forming epidermis, dermal-like compartments, and appendage-like structures
Main cellular composition	Mainly human keratinocytes	Keratinocytes and fibroblasts; some models may include melanocytes or immune cells	Native multicellular skin composition, including keratinocytes, fibroblasts, vascular cells, nerves, immune cells, and appendage-associated cells	iPSC-derived ectodermal and mesodermal lineage cells; can form keratinocytes, dermal-like cells, hair follicle-associated cells, and sebaceous gland-associated cells
Appendages	Absent	Usually absent	Retains native appendages such as hair follicles, sebaceous glands, and sweat glands, but activity is affected by *ex vivo* preservation	Can self-organize to form hair follicle- and sebaceous gland-like structures, but number, maturity, and distribution remain variable
Dosing interface and polarity	ALI culture forms clear apical-basal polarity and is suitable for topical dosing	ALI culture forms relatively clear apical-basal polarity	Native skin orientation is clear and suitable for Franz diffusion cells and IVPT	Classical SkOs are mostly three-dimensional spherical or cystic structures; dosing interface and effective diffusion area require engineering modification, although planarized SkOs may improve this issue
Barrier function	Can form a stratum corneum, but barrier lipid composition still differs from native skin	Closer to full-thickness skin than RHE, but barrier function depends on construction method	Currently remains the reference model closest to the human barrier in IVPT	Has stratum corneum-like structures and tight junction features, but adult-level barrier maturity and standard permeation performance still require systematic validation
Applicability for TDDS evaluation	Suitable for irritation, corrosion, and some preliminary barrier/permeation screening	Suitable for some formulation screening, barrier-damage studies, and dermis-related research	Important reference model for IVPT and percutaneous absorption studies	Suitable for exploring complex TDDS, disease-skin models, appendage-associated delivery, and advanced microphysiological evaluation, but standardized permeation-testing systems are not yet established
Preparation or acquisition time	Commercial models are usually directly available; laboratory construction often requires approximately 2–3 weeks	Usually approximately 3–6 weeks, depending on model complexity	No *in vitro* construction is required, but availability depends on donor source, ethics approval, transport, and storage conditions	Usually requires long-term differentiation culture; formation of mature hair follicle- and sebaceous gland-like structures requires more than approximately 140 days, around 4–5 months
Reproducibility	High; commercial workflows and QC systems are relatively mature	Moderate; affected by cell source, matrix, and construction workflow	Moderate to low; affected by donor age, sex, anatomical site, storage conditions, and batch variability	Currently low to moderate; affected by iPSC line, differentiation efficiency, self-organization variability, and maturity, with insufficient inter-laboratory validation
Relative cost	Low to moderate	Moderate	Moderate to high; depends on tissue source, preservation, and ethical costs	High; mainly due to long-term culture, iPSC maintenance, growth factors, matrix materials, and low throughput
Standardization and regulatory maturity	High; used in OECD TG 431 and TG 439 for skin corrosion/irritation evaluation	Moderate; commercial and research applications exist, but validation scope is less mature than RHE	High reference value, but not a standardized manufactured model; commonly used as an IVPT reference material	Early stage; release criteria, reference chemicals, performance standards, and inter-laboratory validation for TDDS use are still lacking
References	(OECD, 2004; Netzlaff et al., 2005; Kandárová et al., 2006; Ackermann et al., 2010; Lee et al., 2020; Lee et al., 2022; OECD, 2025a, 2025c)			

### 2.2 Regulatory breakthroughs: regulatory change and translational opportunities for skin organoids

The evolution of skin models described above indicates that the central challenge in TDDS evaluation is shifting from whether *in vitro* permeation can be measured to whether more human-relevant, reproducible, and interpretable skin models can be used. This technical need is aligned with recent global regulatory support for non-animal methods and human-relevant models. Regulatory change is increasingly driving drug development and safety assessment away from conventional animal models and toward more human-relevant non-animal approaches. Europe established an early institutional basis for this shift. In 1986, the European Union adopted its first directive on the protection of animals used for scientific purposes. Directive 2010/63/EU later incorporated the principles of replacement, reduction, and refinement (3Rs) into the legal framework and stated that, where an EU-recognized method can provide the required result without using live animals, animal experiments should not be performed (European Commission, 2010). Thus, the EU has not only recently begun to consider non-animal methods, but has long restricted animal experimentation to situations in which acceptable alternatives are not available.

This trend has intensified in recent years. On 1 June 2026, the European Commission formally adopted the Roadmap towards phasing out animal testing for chemical safety assessments, which proposes concrete actions and staged recommendations across regulatory areas to accelerate the transition toward innovative non-animal methods (European Commission, 2026). The roadmap covers multiple safety-assessment contexts, including industrial chemicals, pharmaceuticals, and food additives, and emphasizes that future translation depends not only on reducing animal numbers but also on establishing alternative methods that are validated, acceptable, and suitable for regulatory decision-making. In other words, the European regulatory logic is moving beyond implementation of the 3R principles toward a more systematic pathway for validation, implementation, and regulatory adoption of non-animal methods.

In the United States, the regulatory shift accelerated after 2022. The FDA Modernization Act 2.0 removed the long-standing statutory requirement for animal testing in new drug development and gave cell-based models, computational models, microphysiological systems, and other new approach methodologies (NAMs) clearer institutional space in drug development (U.S. Congress, 2022). In April 2025, the FDA then released a roadmap to reduce animal testing and encouraged the inclusion of NAMs data in IND submissions. The roadmap explicitly mentions AI-based computational models, cell lines, laboratory-grown human organoids, and organ-on-a-chip systems as approaches that may improve human relevance and reduce reliance on animal testing (U.S. Food and Drug Administration, 2025). NIH also announced in 2025 that it would prioritize human-based research technologies and coordinate the development, validation, scaling, and regulatory translation of non-animal methods through ORIVA (National Institutes of Health, 2025).

SkOs are still far from mature TDDS evaluation models. Nevertheless, in a regulatory environment that increasingly encourages non-animal methods and strengthens validation frameworks, they provide a theoretically valuable and forward-looking candidate direction for building more complex and more humanized skin models.

Overall, IVPT has transformed transdermal evaluation from early, dispersed *in vitro* permeation experiments into an *in vitro* assessment system with standardized protocols, quality-control requirements, and regulatory relevance. However, as diffusion devices, receptor conditions, sampling schemes, and statistical methods become increasingly standardized, the major factor limiting IVPT predictivity has gradually shifted from the experimental method itself to the skin model. *Ex vivo* human skin remains the most human-relevant reference material, but it is limited by availability, donor variability, and loss of long-term viability. Animal skin and synthetic membranes are useful for early screening but cannot fully represent the human skin barrier. Engineered models such as RHE offer greater standardization potential but remain limited in dermal architecture, appendageal composition, and complex microenvironmental features. Meanwhile, current regulatory legislation and funding policies do not directly support the use of SkOs for standard TDDS evaluation, but they are encouraging the development of NAMs, organoids, organ-on-a-chip systems, and AI-assisted human-relevant platforms. Further optimization of transdermal evaluation will therefore depend not only on continued refinement of IVPT methods, but also on the establishment and validation of skin models with greater human relevance, structural complexity, and functional interpretability. In this context, SkOs remain far from mature TDDS evaluation models, but they provide a theoretically valuable and forward-looking candidate direction for constructing more complex and more humanized skin models.

## 3 Developmental history of *in vitro* skin models

The development of *in vitro* skin models has not been a simple linear accumulation of techniques. Rather, it has involved a broader shift from cell expansion to tissue reconstruction and, more recently, to organogenesis and system integration. Based on changes in construction strategies and biological complexity, this development can be broadly divided into the following three phases.

### 3.1 Three phases of skin model evolution

#### 3.1.1 Phase I: the era of 2D monolayer cell culture (1906–1980)

This early phase focused mainly on overcoming the basic challenges of cell sourcing and *in vitro* expansion. As early as 1906, Harrison demonstrated that living tissues could be maintained *in vitro* using the hanging drop method (Harrison, 1907). A more substantial advance came in 1975, when Rheinwald and Green established the classic 3T3 feeder-layer co-culture system (Rheinwald and Green, 1975). This work addressed a major limitation in the expansion of primary keratinocytes and enabled large-scale culture *in vitro*. In doing so, it provided an important cell-biological foundation for the later development of more complex three-dimensional skin models.

#### 3.1.2 Phase II: tissue-engineered skin equivalents (1980–2010)

In the 1980s, the introduction of air–liquid interface (ALI) culture significantly advanced the field by moving skin models from the cellular level toward tissue-level reconstruction. Bell et al. first reported the construction of a dermal equivalent based on a collagen lattice, marking an early step in the development of tissue-engineered skin (Bell et al., 1981). Building on this approach, researchers later generated reconstructed human epidermis (RHE) models with stratified architecture, as well as full-thickness skin equivalents, by seeding keratinocytes onto collagen gels or synthetic scaffolds containing fibroblasts under ALI conditions (Pruniéras et al., 1983). As the field matured, Czyz et al. summarized the standardized construction protocols for this generation of models (Czyz et al., 2023). In parallel, efforts were made to reduce donor-related heterogeneity and the limited proliferative lifespan of primary cells. For example, Smits et al. showed that standardized three-dimensional skin models could be generated using immortalized keratinocytes such as the N/TERT cell line, thereby improving reproducibility (Smits et al., 2017). To better reflect the cellular diversity of native skin, advanced models also incorporated melanocytes and immune cells, including Langerhans cells, enabling early *in vitro* reconstruction of pigmentation and basal immune responses (Régnier et al., 1997; Bessou-Touya et al., 1998).

Despite these advances, models from this phase remained fundamentally engineered reconstructions assembled through the layering of different cell types rather than through intrinsic developmental self-organization. This top-down strategy imposed clear physiological and functional limitations. First, the stratum corneum lipid organization of early RHE models, including ceramide subclass distribution, still differed considerably from that of native skin, resulting in a weaker barrier and higher permeability to lipophilic drugs than that observed in normal human skin (Netzlaff et al., 2005). Second, these models lacked complex appendages such as hair follicles, sebaceous glands, and sweat glands, making them unable to reproduce the organogenetic processes and spatial self-organization seen *in vivo*. These limitations gradually shifted attention toward approaches that more closely integrate developmental biology with tissue engineering.

#### 3.1.3 Phase III: self-assembling skin organoids and multi-technology integration (2010–present)

Beginning in the 2010s, *in vitro* skin model research entered a new stage. Progress during this period followed two parallel but conceptually distinct directions, which have increasingly started to converge.

The first direction is a bottom-up, developmentally informed self-assembly strategy that led to the emergence of skin organoids (SkOs). A key step in this area was the 2020 study by Lee et al., who used induced pluripotent stem cells (iPSCs) together with temporally controlled induction by BMP4, TGF-β inhibitors, and FGF2 to model early interactions between the surface ectoderm and cranial neural crest (Lee et al., 2020). Through intrinsic self-organization, the resulting SkOs developed an epidermal–dermal hierarchy and generated appendageal structures such as hair follicles and sebaceous glands. To address the limitations of early cystic SkOs, which restricted topical drug application, De Henau et al. later refined the culture method by introducing an air–liquid interface system that promoted organoid unfolding and planar growth, thereby improving their suitability for conventional transdermal testing (De Henau et al., 2025).

The second direction is a top-down fabrication strategy based on bioengineering, particularly three-dimensional bioprinting and related technologies that enable more precise spatial control over tissue architecture. Lee et al. first demonstrated the feasibility of building complex skin structures through layer-by-layer printing of collagen and cells (Lee et al., 2014). To address the absence of appendages in traditional models, Abaci et al. used 3D-printed molds to generate microwell arrays that spatially constrained dermal papilla cells and promoted hair follicle formation in engineered skin (Abaci et al., 2018). To overcome the vascularization problem that limits the viability of larger tissues, Kim et al. used coaxial extrusion to generate perfusable artificial vascular networks (Kim et al., 2019). Related studies by Baltazar et al. and Mori et al. also demonstrated the construction of vascularized skin grafts and vascular channels on chip-based systems (Mori et al., 2017; Baltazar et al., 2020).

Beyond three-dimensional bioprinting, skin-on-a-chip systems represent another important direction in the development of engineered skin models and are highly relevant to TDDS evaluation. Compared with current SkOs, skin-on-a-chip platforms have a more mature technical basis for fluidic control, dynamic exposure, gradient formation, real-time sampling, and integration of analytical readouts. They may therefore be better suited for dynamic permeation studies and drug exposure monitoring (Bajza et al., 2020; Tárnoki-Zách et al., 2021; Varga-Medveczky et al., 2021). However, skin-on-a-chip systems and SkOs should not be viewed as necessarily competing platforms. The former emphasize engineering control and dynamic analysis, whereas the latter highlight complex skin architecture, appendage formation, disease modeling, and reconstruction of a human-relevant microenvironment. Because direct head-to-head studies comparing SkOs and skin-on-a-chip platforms for TDDS evaluation are still lacking, the two systems are better regarded as complementary platforms rather than models with a clearly established hierarchy.

The most recent trend has been the integration of biological fidelity with engineering control. Microfluidic technologies have given rise to skin-on-a-chip platforms that allow the dynamic simulation of fluid shear stress, drug concentration gradients, and cell migration processes (Wufuer et al., 2016; Varga-Medveczky et al., 2021; Krishnakumar et al., 2023). At the same time, current efforts increasingly aim to combine bottom-up self-assembly with top-down bioengineering. For example, Mostina et al. integrated hiPSC-derived vascular organoids with self-assembled SkOs to generate fully vascularized skin organoids (VSKOs) containing resident immune cell populations (Mostina et al., 2025). These hybrid systems may become valuable tools in future TDDS evaluation. The developmental progression from early culture systems to self-assembling and engineering-integrated skin models, together with representative strategies for further enhancing advanced SkOs, is summarized in Figure 3.

**FIGURE 3:**
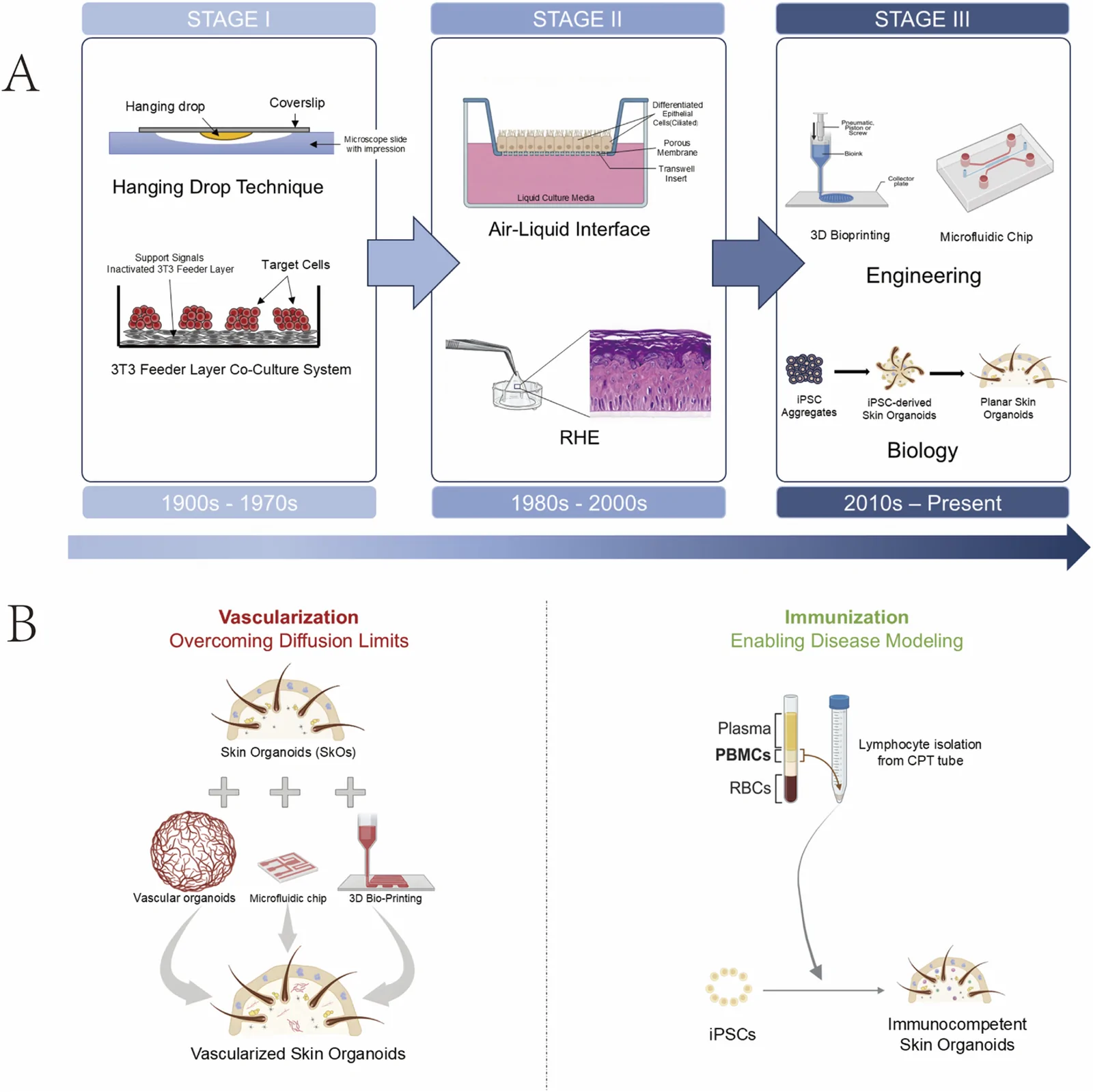
Evolution of *in vitro* skin models and representative strategies to enhance advanced skin organoids. **(A)** Three broad stages in the development of *in vitro* skin models: early two-dimensional cell culture systems, tissue-engineered skin equivalents based on air-liquid interface culture and reconstructed human epidermis (RHE), and the recent convergence of organoid biology with bioengineering approaches, including three-dimensional bioprinting, microfluidic systems, and planar skin organoids. **(B)** Representative strategies to increase the physiological complexity of advanced skin organoids (SkOs), including vascularization through integration with vascular organoids and/or three-dimensional bioprinting, and immune integration through the incorporation of immune-related cellular components to generate more physiologically relevant skin organoid systems.

### 3.2 Physiological characteristics and functional validation of skin organoids

To assess how closely SkOs reproduce the microphysiological features of human skin, researchers have developed a multiscale validation framework spanning cellular composition, tissue architecture, appendage formation, and barrier-related function.

#### 3.2.1 Fidelity of the cellular atlas and tissue architecture

Histologically, SkOs reconstruct the core epidermal–dermal bilayer architecture of human skin through intrinsic self-assembly. Single-cell RNA sequencing data show that SkOs contain major epidermal lineages, including basal stem cells, spinous cells, and granular cells, as well as diverse dermal mesenchymal populations such as fibroblasts, adipocytes, and glial cells (Lee et al., 2020). With ongoing advances in multilineage co-culture, newer SkOs have also shown the capacity to incorporate vascular endothelial cells and macrophages. As a result, these systems are gradually evolving toward more complex microphysiological models with rudimentary immune and metabolic functions (Mostina et al., 2025).

#### 3.2.2 Regeneration and cyclical simulation of skin appendages

One of the most important advances offered by SkOs is their ability to generate complex skin appendages. Current models can form intact hair follicle-like units with concentric organization, together with sebaceous glands capable of lipid secretion; this secretory activity has been supported by BODIPY staining. Xenograft studies have further shown that organoid-derived hair follicles can undergo autonomous anagen–catagen–telogen cycling *in vivo*, a feature that conventional *in vitro* skin models have generally failed to reproduce (Lee et al., 2020). In terms of tissue organization, De Henau et al. reported that planar culture strategies can improve the vertical orientation of hair follicles and make their spatial arrangement more similar to that of native skin. In addition, Shafiee et al. showed that sweat gland-like structures can be induced *in vitro* through targeted manipulation of relevant signaling pathways (Shafiee et al., 2024).

#### 3.2.3 Molecular signatures and developmental stage localization

At the molecular level, Lee et al. showed that dermal cells within SkOs express cranial neural crest-associated markers such as PAX3 and SOX10, consistent with the developmental origin of human facial skin. Gene expression analyses further indicate that the transcriptomic profile of SkOs cultured *in vitro* for approximately 4–5 months resembles that of second-trimester human fetal skin. These findings suggest that SkOs follow key features of normal embryonic skin development rather than representing nonspecific pathological overgrowth (Lee et al., 2020).

#### 3.2.4 Establishment of the physical barrier and biochemical functions

Barrier function is particularly important for TDDS evaluation. Studies have shown that mature SkOs can restrict the permeation of water-soluble tracer dyes such as Lucifer Yellow, indicating the formation of an intact tight-junction network *in vitro*. Transcriptomic analyses also support the expression of key genes involved in lipid metabolism and stratum corneum formation, including FLG, LOR, and IVL (Lee et al., 2020). Although the overall expression profile of current SkOs remains closer to fetal skin than adult skin, De Henau et al. showed that planar ALI culture, compared with conventional suspension culture, can promote the maturation of barrier-related proteins and improve stratum corneum compaction.

Overall, SkOs reproduce important features of native human skin at the levels of tissue architecture, appendage formation, and molecular identity. Although limitations in barrier maturation remain, they currently represent the first high-fidelity three-dimensional *in vitro* model that endogenously includes intact skin appendages. For this reason, SkOs offer a promising platform for investigating transfollicular drug delivery and evaluating complex transdermal formulations.

## 4 Application basis, model advantages, and evidence boundaries of SkOs for TDDS evaluation

Although the potential of skin organoids (SkOs) in transdermal drug delivery evaluation has attracted considerable attention, their practical application in pharmaceutical evaluation is still at an early stage. Compared with the rapid progress in basic biological research, their translational use remains limited. This gap is largely related to the complexity of current fabrication protocols and the lack of mature standardization strategies. Nevertheless, the development of existing commercialized *in vitro* skin models, together with the physiological advantages of SkOs over traditional systems, provides a useful framework for considering how these models may be applied in future transdermal evaluation.

### 4.1 Current application status of SkOs

Since Lee et al. first reported in 2020 that human pluripotent stem cells can be used to generate human skin organoids containing hair follicles, sebaceous glands, and neural-like structures, SkOs have gradually expanded from models of skin development to platforms for disease modeling, drug screening, and regenerative medicine (Lee et al., 2020; Lee et al., 2022). SkOs and related three-dimensional skin models have been used to model viral infection, bacterial infection, genetic skin disorders, inflammatory injury, fibrosis, and scar formation. For example, hiPSC-derived SkOs have been used to establish a SARS-CoV-2 skin infection model, showing that this system can reflect the effects of viral infection on hair follicles, epidermal cells, and neural-related cells (Ma et al., 2022). SkOs have also been used to model cutaneous tuberculosis, revealing fibroblast activation, extracellular matrix remodeling, and fibrosis-related signaling during infection (Yue et al., 2025). In addition, related models have been applied to recessive dystrophic epidermolysis bullosa, KLF4-variant syndromic ichthyosis, and hypertrophic scarring, supporting their potential in modeling genetic, infectious, inflammatory, and fibrotic skin diseases (Garriga-Cerda et al., 2025; Wang et al., 2025; Kim et al., 2026).

Beyond disease modeling, systems related to SkOs are beginning to be used for drug screening and toxicity evaluation. For example, microspheric skin organoids combined with a Wnt/β-catenin reporter system have been used for proof-of-concept drug screening with minoxidil (Xie et al., 2025). Other three-dimensional skin organoids or organoid spheroids have been used to evaluate local nucleic acid delivery, microplastic-induced skin toxicity, and the protective effects of natural active compounds against photoaging (Joseph-Mullol et al., 2025; Guo et al., 2026; Hu et al., 2026). These studies indicate that SkOs may not only model normal skin architecture but also partially reconstruct disease- or injury-associated skin microenvironments. Disease-oriented SkOs may therefore provide a more pathologically relevant human model basis for evaluating topical or transdermal delivery in psoriasis, atopic dermatitis, skin infection, scarring, and genetic barrier defects.

However, our literature search did not identify clear studies in which Lee-type hiPSC-derived SkOs were used as standardized skin barrier models in Franz diffusion cells or conventional *in vitro* permeation testing (IVPT), with systematic measurement of quantitative permeation parameters such as cumulative permeation, steady-state flux, permeability coefficient, or skin retention. Thus, the current role of SkOs in TDDS evaluation remains largely at the level of disease modeling and potential alternative platforms, rather than that of a mature pharmaceutic permeation model. If planarized, barrier-mature, size-compatible, and batch-stable platforms based on SkOs can be established and correlated with classical IVPT systems and *ex vivo* human skin data, SkOs may open new opportunities for topical delivery, transdermal delivery, and personalized permeation evaluation under disease-relevant skin conditions.

### 4.2 Commercialization as a foundation: lessons from the success of RHE for the translation of SkOs

Before discussing the future application of SkOs, it is useful to revisit the commercialization pathway of reconstructed human epidermis (RHE) and full-thickness skin models. Although RHE is not an organoid system in the strict sense, its success as a human-derived *in vitro* skin model provides an important reference for translating SkOs from basic research models into usable evaluation platforms. The development of RHE shows that the value of human *in vitro* models lies not only in replacing animal experiments, but also in providing higher human relevance, better production consistency, and more industrially compatible workflows within clearly defined contexts of use.

Compared with animal skin, the core advantages of RHE are its human origin and its capacity for standardized production. Animal skin differs from human skin in stratum corneum thickness, follicle density, lipid composition, and metabolic features, all of which can affect extrapolation of irritation, corrosion, and drug permeation results. Commercial RHE models such as SkinEthic™ RHE and EpiDerm™ are constructed from human keratinocytes and can form a stratified epidermis and functional stratum corneum. On the basis of OECD TG 431 and TG 439, these models are now widely used for *in vitro* skin corrosion and irritation testing and have become important alternatives to animal testing in relevant settings (Kandárová et al., 2006; OECD, 2025b).

The success of RHE and full-thickness skin models is not due solely to morphological similarity, but also to mature systems for production, transport, quality control, and endpoint readout. Quality control indicators such as TEER, ET50, tissue viability, barrier integrity, and tissue morphology help maintain batch-to-batch functional consistency and improve data comparability across laboratories (Faller et al., 2002). This experience has direct implications for SkOs. Although SkOs are structurally more complex than RHE and can form hair follicles, sebaceous glands, and dermal-like compartments, complexity alone is not sufficient for translation. If SkOs are to be used in TDDS evaluation, their development should emphasize not only physiological structure but also manufacturability, quality control, reproducibility, and comparability.

### 4.3 Overcoming physiological limitations: core advantages and evidence boundaries of SkOs in transdermal drug delivery evaluation

Animal skin, *ex vivo* human skin, and RHE have long supported *in vitro* TDDS evaluation, but each model has important limitations. Animal skin is affected by species differences, RHE cannot fully reproduce full-thickness skin and appendageal structures, and *ex vivo* human skin is limited by tissue availability, donor variability, ethical requirements, and declining viability. In contrast, SkOs can self-organize *in vitro* to form stratified epidermis, dermal-like structures, hair follicles, sebaceous glands, Merkel cells, and sensory neuron-like structures. They therefore provide a new opportunity to build more complex human-relevant skin models (Lee et al., 2020; Lee et al., 2022).

It should be emphasized that these biological properties do not mean that SkOs can already serve as standard TDDS evaluation models. Current evidence mainly supports the potential of SkOs in skin development research, disease modeling, appendage formation, planar culture, vascular or immune integration, and drug screening. Direct validation remains limited in classical TDDS settings, especially Franz diffusion cells, IVPT, steady-state flux, permeability coefficient, cumulative permeation, and skin retention. Thus, SkOs are best viewed as promising next-generation candidate models rather than mature standard substitutes.

#### 4.3.1 Recapitulation of penetration pathways and appendageal advantages

Percutaneous absorption occurs mainly through intercellular, transcellular, and appendageal routes. RHE can partially mimic the epidermal barrier but usually lacks hair follicles, sebaceous glands, and sweat glands. Animal skin contains appendages, but follicle density, stratum corneum structure, lipid composition, and skin thickness differ from human skin. Studies have shown that hiPSC-derived SkOs can form stratified epidermis, dermal-like structures, hair follicles, and sebaceous glands, and can partially recapitulate the cellular composition and tissue architecture of fetal-stage skin (Lee et al., 2020; Lee et al., 2022). Planarized and air-liquid interface culture methods have further improved the limitations of cystic SkOs for topical application, making them closer to planar systems suitable for *in vitro* skin research (De Henau et al., 2025; Sun et al., 2025).

At present, however, there is insufficient evidence that appendageal structures in SkOs have been directly used to quantify appendageal flux, follicular reservoir effects, or follicle-mediated nanocarrier delivery in TDDS studies. The appendageal structures of SkOs provide an important biological basis for studying complex delivery routes, but whether they can stably and quantitatively reflect drug permeation through different pathways still requires validation in standard IVPT systems and comparison with *ex vivo* human skin.

#### 4.3.2 Barrier maturity

Another potential advantage of SkOs is their human origin and multicellular self-organization. Compared with animal skin, pluripotent stem cell-derived SkOs are closer to human skin in genetic background and cellular origin. Compared with RHE, SkOs can form more complex epidermal-dermal-like structures and appendageal systems. Existing studies show that SkOs can express markers related to epidermal differentiation, keratinization, and appendage formation (Lee et al., 2020; Lee et al., 2022; Sun et al., 2025).

For TDDS evaluation, however, the key question is not simply whether SkOs “look like skin”, but whether their barrier function is sufficiently mature and stable to generate reproducible permeation data. Systematic comparisons remain limited for SkOs stratum corneum lipid composition, ceramide subclass distribution, tight junction maturity, TEER, dye permeability, drug flux, and skin retention. In particular, whether SkOs can accurately predict permeation behavior of compounds with different physicochemical properties compared with adult *ex vivo* human skin has not been fully validated. Human origin and structural complexity are therefore prerequisite advantages, but they do not by themselves establish standard permeation-testing capability.

#### 4.3.3 Potential value for physical penetration enhancement technologies

The development of microneedles, iontophoresis, electroporation, ultrasound, and thermal ablation means that *in vitro* skin models must not only provide barrier function, but also reflect skin biomechanics. Microneedle insertion depth, fracture risk, skin recoil, local tissue deformation, and drug release kinetics are closely related to dermal architecture, collagen matrix, and viscoelasticity. RHE offers limited support for these assessments because it lacks a complete dermal compartment.

SkOs contain fibroblasts and a dermal-like matrix, and their early morphogenesis is influenced by mechanical force-mediated mesenchymal–epithelial interactions, including dermal cell contraction, cytoskeletal regulation, calcium signaling and epidermal Piezo1 activation (Wang et al., 2023). This suggests that SkOs may provide a more complex *in vitro* basis for studying skin tissue mechanics and physical enhancement technologies. However, we did not identify original studies directly using SkOs for microneedle insertion, puncture-depth measurement, mechanical stability testing, or post-enhancement drug release kinetics. This application therefore remains a plausible hypothesis rather than a validated use case and will require mechanical and permeation comparisons with *ex vivo* human or animal skin.

#### 4.3.4 Vascular, immune, and neural integration: from barrier models to disease-relevant skin microphysiology

A long-standing limitation of traditional *in vitro* skin models is the lack of vascular, immune, and neural components, which are important for drug delivery and local responses in diseased skin. Inflammatory skin diseases, infectious skin diseases, scarring, and barrier-defect disorders not only alter stratum corneum permeability, but also affect the dermal microenvironment, immune-cell infiltration, vascular responses, and neuroimmune regulation. Simple barrier models therefore cannot fully capture topical or transdermal delivery under disease conditions.

Recent studies have attempted to increase the physiological complexity of SkOs. Mostina et al. generated vascularized skin organoids and observed integration of endothelial cells, mural cells, hematopoietic or immune-related cells, and mesenchymal cells into skin structures (Mostina et al., 2025). In neural aspects, Lee et al. reported sensory neuron-like structures, Schwann cells, and neural-like networks targeting Merkel cells in SkOs, while Yuan et al. further used human pluripotent stem cell-derived SkOs to investigate Merkel cell generation and IGF1R signaling (Lee et al., 2020; Yuan et al., 2026).

These advances indicate that SkOs are evolving from structural mimicry toward more complex neuroimmune and vascularized skin microphysiological models. For TDDS research, this trend may shift transdermal evaluation beyond the question of whether a drug crosses the stratum corneum, toward how a drug distributes in diseased skin, whether it induces local inflammation or irritation, whether it affects dermal-cell responses, and whether it can exert therapeutic effects in specific pathological microenvironments. However, these advanced SkOs systems have so far mainly been used for skin development, disease modeling, inflammatory responses, and tissue engineering, rather than standard TDDS permeation evaluation. Their current significance is therefore better framed as providing a model basis for next-generation disease-skin drug-delivery evaluation.

#### 4.3.5 SkOs provide a new model basis for TDDS evaluation

At present, different SkOs construction strategies exhibit distinct morphological and functional advantages. For example, classical hiPSC-derived SkOs show relatively high complexity in the formation of hair follicles, sebaceous glands and neural-like structures; flattened or air-liquid interface-cultured SkOs may be more compatible with topical application and barrier exposure; miniaturized or microspherical SkOs may be more suitable for drug screening and scalable operation; and vascularized or immune-integrated SkOs may be better suited for modeling disease-related skin microenvironments and local tissue responses. However, these advantages are currently inferred mainly from structural phenotypes, cellular composition or disease-modeling capacity, and have not been validated under unified standards for TDDS evaluation. In particular, head-to-head comparisons among different SkOs protocols are still lacking, as are systematic comparisons with *ex vivo* human skin, RHE or other engineered skin models in terms of steady-state flux, permeability coefficient, lag time, skin retention and barrier integrity. Therefore, it is currently not possible to determine which SkOs protocol performs “best” for drug permeation testing. For TDDS research, the value of different SkOs platforms should still be understood as theoretical model fitness rather than experimentally proven superiority. Future studies will need to compare epidermal maturation, stratum corneum lipid organization, effective diffusion area, batch-to-batch reproducibility, IVPT compatibility and correlation with *ex vivo* human skin data under unified quality-control standards before the true applicability of SkOs in transdermal drug delivery evaluation can be defined.

Overall, SkOs have demonstrated features that traditional skin models rarely combine, including human origin, multicellular self-organization, stratified epidermis, dermal-like structures, hair follicles, sebaceous gland-like appendages, and the potential for further vascular, immune, and neural integration. These features make SkOs useful not only for studying complex skin biology and disease modeling, but also for providing a model basis that may more closely reflect the human skin microenvironment in future local and transdermal drug delivery evaluation. Their value is especially compelling in settings that conventional models cannot fully cover, including diseased skin, appendage-associated delivery, long-term local responses, and individualized drug delivery assessment.

## 5 Translational bottlenecks of skin organoids in transdermal drug delivery evaluation

Although SkOs have clear advantages over traditional models in recapitulating complex skin architecture and some physiological functions, their practical application in transdermal drug delivery evaluation remains at an early stage. No clear reports have yet systematically used SkOs to measure TDDS permeation parameters. More importantly, current SkOs still show batch-to-batch variability, structural heterogeneity, and inconsistent maturation. Before these critical quality attributes are stabilized, permeation flux, permeability coefficient, or skin-retention data may be difficult to interpret, reproduce, and compare across laboratories. Thus, the main obstacle to translating SkOs into TDDS evaluation platforms is not only the lack of permeation data, but also the absence of standardized biomanufacturing and quality control systems capable of generating reliable data.

### 5.1 Lack of standardization in production and quality control

For any drug evaluation platform, regulatory acceptance depends not only on structural similarity to human tissue, but also on the ability to generate reproducible, interpretable, and comparable data. Current SkOs generation still relies heavily on stem-cell self-organization in three-dimensional culture, a process with considerable morphogenetic uncertainty that can lead to batch-to-batch and within-batch heterogeneity. iPSC-derived models are particularly sensitive to subtle changes in the initial microenvironment, which can significantly affect differentiation trajectories and final tissue phenotypes (Volpato and Webber, 2020). Even within the same batch, parallel SkOs may differ in size, epidermal thickness, hair follicle number, tissue polarity, and maturity.

This variability is especially important for TDDS evaluation. If barrier maturity, effective diffusion area, tissue polarity, appendage distribution, and batch consistency are not standardized, measured permeation parameters may be difficult to attribute to formulation performance rather than differences in SkOs structure or maturity. In contrast, commercial RHE models have established more mature manufacturing and quality control frameworks that support data comparability across laboratories. For SkOs, widely accepted release criteria for barrier integrity, lineage composition, tissue morphology, maturation stage, appendage number, polarity, and functional readouts have not yet been established (Huch et al., 2017). The first step in translating SkOs into TDDS evaluation should therefore be to establish reproducible manufacturing and quality control systems rather than to directly pursue permeation-parameter measurement.

### 5.2 Structural barriers of economic cost and time efficiency

Whether a model can be adopted at scale in drug development depends not only on biological relevance, but also on cost, timeline, and data-output efficiency. Although SkOs have high human relevance and structural complexity, current fabrication workflows remain time-consuming, costly, and technically demanding, making them poorly aligned with the rapid iteration, high throughput, and low cost required in TDDS development.

From a time perspective, SkOs generation is a slow morphogenetic process. Lee et al. reported that differentiation of human iPSCs into SkOs containing mature hair follicle- and sebaceous gland-like structures typically requires more than 140 days of continuous culture (Lee et al., 2020). In contrast, two-dimensional cell models can usually be established within days, and conventional RHE models are often ready for experimentation within 2–3 weeks. Such a prolonged timeline increases labor, material, and maintenance costs and also raises the risk of contamination, culture fluctuation, and technical failure.

Economic cost further limits translation. Current SkOs culture systems usually depend on recombinant factors such as BMP4 and FGF2, small-molecule regulators, and three-dimensional matrices. Matrigel can support organoid formation, but it is expensive and derived from tumor extracts, creating batch-to-batch compositional variability and standardization challenges (Aisenbrey and Murphy, 2020). In addition, the effective surface area of SkOs is typically small and does not directly meet the requirements of Franz diffusion cells or conventional IVPT devices for continuous, flat, large-area skin samples. From a physical loading perspective, standard IVPT usually requires a continuous skin membrane at the centimeter scale that can be sealed and has a defined barrier orientation. For example, the FDA IVPT draft guidance provides a diffusion-cell example with an effective dosing area of approximately 1.77 cm^2^. By contrast, the effective exposure area of SkOs is currently at the millimeter scale, and its three-dimensional architecture, tissue polarity, and surface continuity remain difficult to reconcile with the skin-membrane loading requirements of conventional Franz diffusion cells or IVPT systems.

At present, SkOs are therefore more suitable as advanced human-relevant models for mechanistic studies, disease modeling, and validation of complex delivery scenarios than as immediate replacements for RHE, *ex vivo* skin, or animal skin in routine large-scale TDDS screening. Future progress will require shortened culture cycles, lower production costs, chemically defined matrices, expanded effective testing areas, and improved automation.

### 5.3 Limitations in biological maturity, iPSC line variability, and genetic stability

Although SkOs can form stratified epidermis, dermal-like mesenchyme, hair follicles, sebaceous glands, and other appendageal structures, their developmental stage and functional maturity may still differ from adult skin. Pluripotent stem cell-derived organoids often recapitulate developmental processes *in vitro* rather than fully mature adult organ states (Clevers, 2016). This is particularly important for TDDS evaluation because drug permeation depends strongly on stratum corneum maturation, lipid lamellar organization, ceramide composition, tight-junction integrity, and epidermal turnover. Systematic permeation evidence comparing SkOs barrier maturity with adult *ex vivo* human skin is still lacking. Thus, the possibility that SkOs may overestimate drug permeation remains primarily an inference from barrier biology rather than a direct conclusion from standard TDDS experiments.

Differences among iPSC lines are another critical issue for SkOs translation. Large-scale iPSC studies have shown that line-to-line variability is driven by donor genetic background, reprogramming, epigenetic state, and culture history. The HipSci project analyzed 711 iPSC lines from 301 healthy donors and found that 5%–46% of variation in iPSC phenotypes, including differentiation capacity and cellular morphology, could be attributed to inter-individual differences (Kilpinen et al., 2017). Kyttälä et al. further showed that donor genetic background can influence iPSC expression profiles, methylation status, and differentiation potential (Kyttälä et al., 2016). Single-cell studies also indicate that different iPSC lines can show distinct cell-state transition trajectories during differentiation (Cuomo et al., 2020).

Initial evidence for this issue has already appeared in SkOs research. Shafiee et al. used three different hiPSC lines to generate SkOs. Although all three lines formed complex organoids with stratified skin structures and pigmented hair follicles, their generation efficiencies differed (Shafiee et al., 2024). This suggests that iPSC line variability may affect SkOs construction efficiency, cellular composition, and tissue maturity. For TDDS evaluation, if SkOs generated from different lines differ in keratinization, lipid barrier, appendage density, or tissue polarity, the same formulation may yield different flux, apparent permeability coefficient, or skin-retention values. Such variability would weaken the reliability of comparisons across laboratories.

Genetic stability also requires attention. During reprogramming, clonal expansion, and long-term passaging, iPSCs may acquire chromosomal abnormalities, copy-number variants, or somatic mutations that affect proliferation, differentiation propensity, and tissue phenotype (Gore et al., 2011; Hussein et al., 2011; Taapken et al., 2011). If SkOs are to become high-quality, scalable TDDS evaluation models, standardized iPSC banks and reference lines will be needed, together with validation of unified differentiation protocols across multiple reference lines. Quality control should also extend beyond routine karyotyping, CNV analysis, mutation screening, and pluripotency-marker testing to TDDS-oriented release criteria, including epidermal thickness, stratum corneum maturity, tight-junction integrity, lipid composition, ceramide profile, appendage density, tissue polarity, effective diffusion area, and baseline barrier-function readouts.

### 5.4 Methodological quality and evidence maturity assessment

The evidence included in this review is methodologically heterogeneous, including narrative reviews, *in vitro* skin models, animal studies, *ex vivo* human skin permeation experiments, SkOs construction studies, and AI prediction models. We therefore did not apply a single risk-of-bias tool uniformly across all references. Instead, we considered study-type-specific appraisal frameworks. SANRA can inform appraisal of narrative review structure; AMSTAR 2 and ROBIS are applicable to systematic reviews; SYRCLE and ARRIVE 2.0 are relevant to animal studies; and ToxRTool and SciRAP can support evaluation of toxicological and *in vitro* research. For skin permeation studies, IVPT-specific quality elements are particularly important, including skin source, barrier integrity, diffusion-cell conditions, receptor-fluid sink condition, drug recovery, analytical-method validation, and experimental reproducibility. For AI prediction models, PROBAST + AI can be used to assess risk of bias and applicability, whereas TRIPOD + AI or CLAIM are more appropriate for reporting completeness. Table 2 summarizes the relevant appraisal frameworks and our qualitative assessment of evidence maturity across the main evidence categories.

**TABLE 2:** Methodological quality and evidence maturity of key evidence supporting SkOs as potential TDDS evaluation platforms.

Evidence type	Representative evidence/study type	Applicable appraisal framework	Main appraisal dimensions	Evidence maturity	Implication for this review
RHE and commercial full-thickness skin models	OECD-validated skin irritation/corrosion models; commercial RHE and full-thickness skin models	OECD validation principles; ToxRTool/SciRAP	Protocol standardization, batch consistency, barrier function, inter-laboratory transferability, defined applicability domain	High	Shows that human *in vitro* skin models can gain regulatory and industrial acceptance after standardization, providing a reference for translation of SkOs
*Ex vivo* human skin and IVPT studies	OECD TG 428; *in vitro* percutaneous absorption and IVPT studies	IVPT-specific quality criteria; OECD/FDA-related principles	Donor source, anatomical site, skin thickness, integrity testing, receptor-fluid sink condition, recovery, sampling design, analytical-method validation	High to moderate	Remains an important reference standard for TDDS permeation evaluation, but is limited by tissue availability, donor variability, and loss of long-term viability
Basic studies on SkOs construction	Original studies generating hair follicles, sebaceous glands, and multilineage skin structures from iPSC-derived SkOs	*In vitro* model reliability/QC principles; SciRAP/ToxRTool-related dimensions	Lineage composition, tissue architecture, maturity, batch variability, culture time, reproducibility, and release criteria	Moderate to low	Supports the ability of SkOs to reproduce complex skin architecture, but is not sufficient to prove direct suitability for standard TDDS permeation testing
SkOs disease models and drug-response studies	SkOs models related to inflammatory skin diseases, viral infection, and genetic skin disorders	Reliability and relevance appraisal for *in vitro* studies; QUIN used cautiously only when applicable	Clarity of disease phenotype, control design, reproducibility, drug-response readouts, and consistency with patient skin features	Moderate to low	Suggests that SkOs can model diseased skin and may support future evaluation of topical or transdermal delivery under disease conditions
SkOs for TDDS permeation evaluation	Clear reports of standard IVPT/Franz diffusion-cell permeation-parameter studies are currently lacking	IVPT quality criteria; definition of model applicability domain	Effective diffusion area, barrier integrity, tissue polarity, permeation flux, permeability coefficient, skin retention, batch consistency	Low/evidence gap	Represents a key gap identified in this review. At present, SkOs are better viewed as frontier potential models rather than validated standard TDDS evaluation platforms
Animal skin and animal experiments	Mouse, porcine, and other animal skin models, including *in vivo* and *ex vivo* experiments	SYRCLE risk-of-bias tool; ARRIVE 2.0 reporting guidelines	Randomization, blinding, allocation concealment, animal information, sample size, exclusion criteria, completeness of outcome reporting	Moderate	Provides biological and pharmacokinetic reference information, but species differences limit direct extrapolation to human skin permeation
AI/CV-based organoid quality control	Applications of computer vision and machine learning in image analysis of other organoids or skin models	PROBAST + AI; TRIPOD + AI; CLAIM	Representativeness of training data, external validation, overfitting risk, model interpretability, reporting completeness, clinical or experimental applicability	Low to moderate	Most evidence comes from other organoids or skin imaging platforms and remains transferable but insufficiently validated for SkOs-TDDS.
Mechanistic permeation models, PBPK, and digital twin frameworks	Mechanistic skin permeation models, PBPK models, microneedle finite-element simulations, and conceptual digital twin frameworks	Model-validation principles; predictive-model reporting standards; applicability-domain assessment	Parameter sources, calibration methods, independent validation, prediction error, model interpretability, and connection with experimental data	Moderate to low	Can support TDDS prediction and experimental design, but should not be equated with validated SkOs experimental platforms
(OECD, 2004; Schneider et al., 2009; Hooijmans et al., 2014; Whiting et al., 2016; Shea et al., 2017; Baethge et al., 2019; Mongan et al., 2020; Percie du Sert et al., 2020)					

This tiered appraisal indicates that the evidence maturity is relatively high for RHE and *ex vivo* human skin, whereas SkOs evidence is concentrated mainly in tissue morphogenesis, disease modeling, and drug-response observation. Direct evidence supporting SkOs as standard TDDS permeation-evaluation platforms remains insufficient. Thus, our assessment of the value of SkOs in TDDS should be understood as a forward-looking interpretation based on current structural and functional evidence, rather than as a conclusion that regulatory validation has already been achieved.

Overall, the translation of SkOs into TDDS evaluation is limited not by a single technical issue, but by a systemic set of bottlenecks involving biomanufacturing, quality control, model maturity, experimental compatibility, evidence level, and regulatory validation. Existing studies have shown that SkOs can reproduce complex skin architecture, appendage formation, and disease-related phenotypes, but these advantages have not yet been fully converted into reproducible, quantitative, and cross-laboratory-comparable permeation-evaluation capability. At present, SkOs are therefore better positioned as advanced human-relevant models for disease modeling, mechanistic exploration, and validation of complex delivery scenarios rather than as mature platforms that can directly replace RHE, *ex vivo* human skin, or standard IVPT systems. Future incorporation of SkOs into TDDS evaluation will require progress in manufacturing workflows, release criteria, engineered formats, barrier validation, and data-prediction frameworks.

## 6 Digital intelligence for standardizing and predicting SkOs-TDDS evaluation?

Improving the standardization of SkOs in transdermal drug-delivery evaluation cannot rely solely on culture-condition optimization and biomanufacturing workflows. Computer vision (CV), machine learning (ML), microfluidic control, and digital twin technologies may provide new methodological support for organoid quality control, model stratification, and transdermal absorption prediction. However, direct evidence for these technologies in SkOs and SkOs-TDDS evaluation remains limited. Existing studies can be broadly grouped into three evidence levels. The first includes digital quantification and permeation-evaluation applications already reported in skin models, skin equivalents, or skin-on-a-chip platforms. The second includes AI-based image analysis and automated culture methods validated in other organoid systems but still requiring transfer to SkOs. The third includes physiologically based pharmacokinetic (PBPK) models, mechanistic permeation models, inverse modeling, microneedle simulation, and digital twin concepts that have not yet been directly coupled to SkOs experimental data. Accordingly, this section discusses how digital intelligence tools may empower SkOs-TDDS platforms in quality control, model standardization, and multiscale prediction, and how they may accelerate the transition of SkOs from Frontier biological models toward translatable evaluation systems. Figure 4 illustrates a conceptual framework for AI-assisted quality control and potential digital twin applications in SkOs-based TDDS evaluation.

**FIGURE 4:**
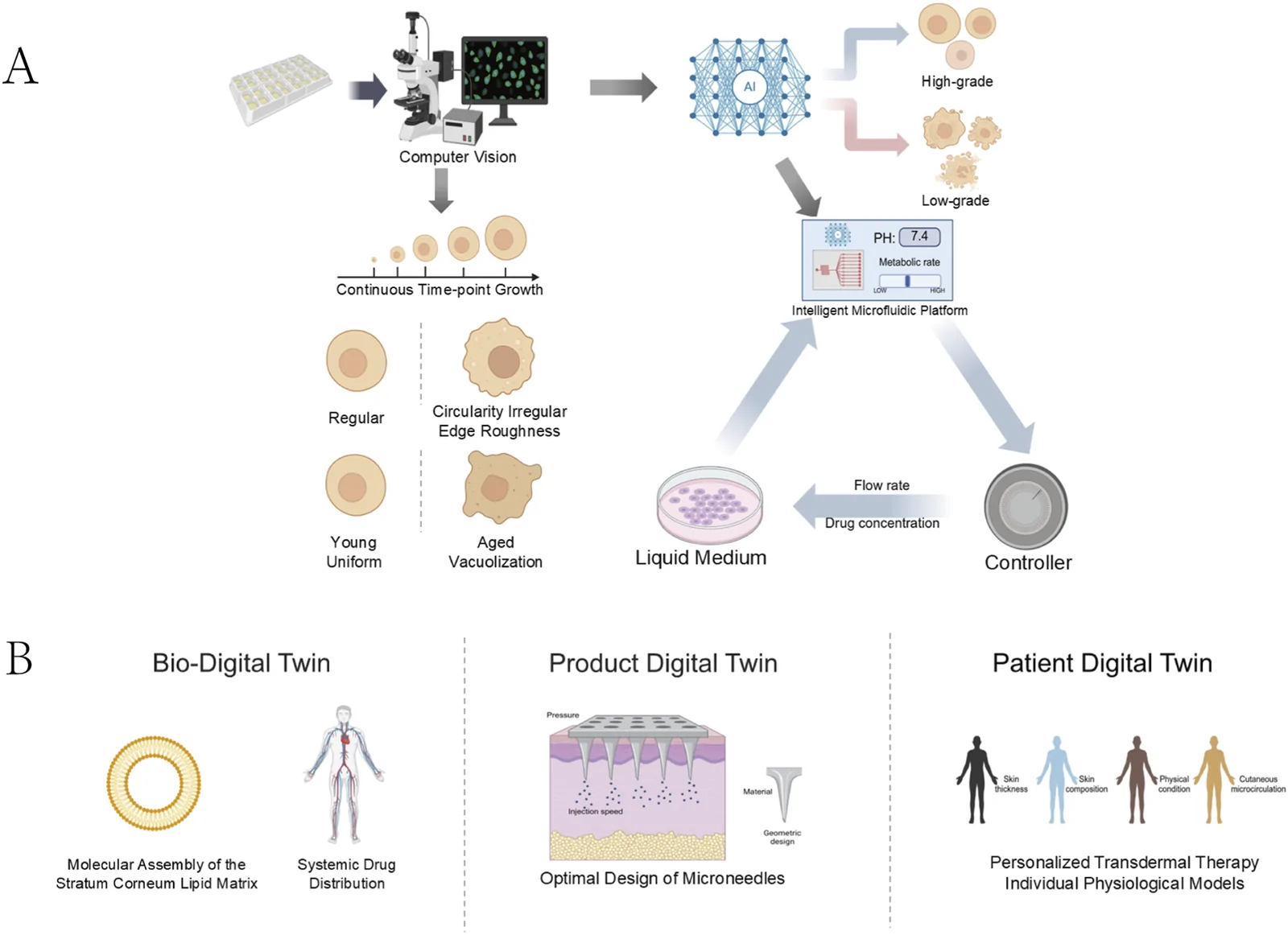
AI-enabled quality control and digital twin frameworks for next-generation TDDS evaluation. **(A)** Conceptual workflow showing how computer vision and artificial intelligence (AI) may support non-invasive skin organoid phenotyping, longitudinal growth tracking, morphological quality grading, and closed-loop regulation of microfluidic culture conditions. **(B)** Potential digital twin applications at the biological, product, and patient levels, including simulation of stratum corneum lipid organization and systemic drug distribution, optimization of transdermal product design such as microneedles, and personalized transdermal therapy based on individual physiological characteristics and disease-related skin states.

### 6.1 Digital quantification in skin-related systems: limited direct evidence but a clear direction

In skin-related models, digital tools have begun to support objective quantification of tissue morphology and barrier-associated features. For example, automated image analysis based on optical coherence tomography (OCT) has been used to measure epidermal thickness in human skin equivalents, showing that non-destructive imaging can support quantitative quality control of skin models (Sanchez et al., 2022). Human skin imaging studies have also shown that ML can extract structural features associated with psoriasis, aging, or microvascular disease from optoacoustic mesoscopy images (He et al., 2024). These studies are not SkOs studies, but they show that morphology- and function-related information in skin tissue can be captured by imaging data and algorithmic models.

More directly related to TDDS are skin-on-a-chip and skin microphysiological systems. Existing studies indicate that skin-on-a-chip platforms can model skin barrier properties, perfusion environments, and transdermal delivery processes, and may be useful for screening transdermal drug or cosmetic absorption (Varga-Medveczky et al., 2021; Govey-Scotland et al., 2023). These platforms provide engineering references for SkOs translation, particularly for microfluidic perfusion, controlled dosing, real-time sampling, and barrier monitoring. However, this evidence mainly comes from skin chips, reconstructed skin, or skin equivalents and cannot be directly equated with mature SkOs-TDDS applications.

### 6.2 Organoid AI-QC technologies as a reference for SkOs quality control

Compared with SkOs, evidence for AI and CV is more extensive in other organoid systems. OrganoID can track individual organoids in microscopy images and quantify size, growth rate, and morphological features (Matthews et al., 2022). D-CryptO also demonstrated that deep learning can analyze morphology from bright-field images of colon organoids, reducing dependence on destructive staining and manual judgment (Abdul et al., 2022). Similar tools have been used for organoid image segmentation, morphological analysis, and drug-screening workflows (Wang et al., 2022; Park et al., 2023). These studies provide a transferable methodological basis for SkOs quality control.

For SkOs, the potential value of these approaches lies in converting manual microscopic inspection into a more objective batch-stratification system. In principle, CV could identify SkOs size, roundness, epidermis-like stratification, tissue polarity, number of hair follicle-like structures, appendage distribution, and necrotic regions, and could exclude structurally abnormal or insufficiently mature samples before TDDS experiments. Automated organoid platforms also show that engineered culture and automated handling can improve within-organoid consistency while preserving patient-to-patient differences (Jiang et al., 2020).

This extrapolation must nevertheless remain cautious. Image features from intestinal, colonic, or tumor organoids are not equivalent to SkOs barrier-function features. For TDDS evaluation, a “morphologically good” organoid does not necessarily have a mature stratum corneum, intact lipid barrier, or stable permeation behavior. SkOs-specific AI-QC therefore needs to be linked to functional readouts, including TEER, tracer permeation, drug flux, skin retention, lipid composition, tight junction protein expression, and appendage density. Only when image features can predict these barrier-function indicators can AI-QC become a release tool for SkOs-TDDS evaluation.

### 6.3 From PBPK to digital twins: the potential value of SkOs data in multiscale prediction

Discussions of digital twins require particular attention to technological maturity. PBPK models, mechanistic skin permeation models, inverse modeling, microneedle finite-element simulation, and digital twins are not the same type of technology. PBPK and mechanistic permeation models have already been applied in topical and transdermal formulation research. For example, *in vitro* skin permeation data have been combined with PBPK modeling to extrapolate *in vivo* exposure for topical metronidazole formulations (Arora et al., 2022). PBPK has also been used to support discussions of bioequivalence and regulatory evaluation for topical products such as diclofenac sodium topical gel (Tsakalozou et al., 2021). These models are relatively mature and can connect *in vitro* release, skin permeation, intradermal distribution, and systemic exposure.

Inverse mechanistic modeling is better understood as a tool for parameter identification and model calibration rather than as a complete digital twin. The inverse mechanistic modeling strategy proposed by Defraeye et al. can identify key parameters in transdermal delivery models and reduce the difficulty of calibrating complex models (Defraeye et al., 2021). If SkOs can provide data such as effective diffusion area, epidermal and stratum corneum thickness, cumulative permeation, steady-state flux, lag time, skin retention, and disease-state barrier changes, these data could serve as parameter priors, calibration inputs, and validation benchmarks for PBPK or mechanistic permeation models.

Microneedle simulation is mainly a computational tool at the product and device level. Finite-element analysis has been used to study the mechanical behavior of microneedle arrays during insertion into skin, including skin deformation, puncture processes, and structural design parameters (Shu et al., 2021). Studies of three-dimensional printed microneedles have also combined finite-element analysis with *in vitro* human skin delivery experiments to evaluate microneedle geometry and penetration performance (Xenikakis et al., 2019). These studies show that computational simulation can reduce physical prototyping and guide optimization of microneedle materials, geometry, and insertion parameters. At present, however, such models mainly rely on *ex vivo* skin or simplified mechanical parameters and have not been systematically coupled to SkOs biomechanics, appendage distribution, or barrier-maturity data.

A digital twin in the strict sense should be a higher-level dynamic virtual system that integrates individualized data, mechanistic models, real-time or longitudinal data streams, and model-updating strategies (Björnsson et al., 2019; Laubenbacher et al., 2022). A single PBPK model, one microneedle finite-element simulation, or a static ML predictor should therefore not be directly described as a complete digital twin. For SkOs-TDDS, future digital twins are more likely to take the form of integrated frameworks. SkOs would provide human-relevant, disease-associated, and formulation-response data; PBPK and mechanistic permeation models would describe drug transport; microneedle or patch simulations would describe product-level release, insertion, and material behavior; and ML would update parameters from imaging, omics, and permeation data to predict delivery performance under untested conditions.

The value of SkOs within a digital twin framework is therefore not to replace existing computational models, but to provide experimental inputs that are difficult to obtain consistently from RHE, animal skin, or *ex vivo* skin. These inputs may include tissue-structure parameters, barrier-function parameters, lipid and tight junction-related molecular indicators, disease-state barrier changes, and delivery differences among formulations or enhancement systems in healthy and diseased SkOs. Only when such data are continuously coupled to computational models can SkOs move from single *in vitro* models toward components of multiscale TDDS prediction platforms.

Overall, digital intelligence tools do not mean that the standardization problems of SkOs-TDDS platforms have already been solved. Rather, they may become important accelerators that help move this platform from concept toward application. Current evidence indicates that quantitative imaging, AI-based image analysis, automated culture, skin-on-a-chip engineering experience, PBPK modeling, inverse modeling, and microneedle simulation can contribute to model quality control, experimental design, and transdermal absorption prediction. Although SkOs-specific AI quality control, closed-loop culture control, and SkOs data-driven digital twins still require validation, these tools may help address current limitations in batch heterogeneity, quality stratification, experimental throughput, and data interpretation. More importantly, the human origin, appendageal architecture, disease-modeling capacity, and potential personalized derivation of SkOs make them highly attractive candidates for future TDDS evaluation. With AI-assisted quality control, automated manufacturing, and multiscale predictive modeling, SkOs may gradually expand from disease-modeling tools into a new generation of human-relevant models supporting topical delivery, transdermal delivery, and formulation evaluation under complex skin conditions.

## 7 Discussion

The evolution of transdermal drug delivery evaluation systems reflects a gradual shift from conventional experimental models toward platforms with greater physiological relevance and stronger technical integration. Within this transition, skin organoids (SkOs) stand out because they can reproduce appendageal structures such as hair follicles and sebaceous glands, thereby providing a more relevant framework for studying transdermal absorption pathways *in vitro*. These characteristics suggest that SkOs may become an important human-relevant platform for evaluating advanced transdermal delivery systems.

At the same time, the translational application of SkOs remains constrained by several important limitations, including incomplete functional maturation, manufacturing complexity, limited standardization, and insufficient compatibility with large-scale screening workflows. Continued advances in stem cell bioengineering, automation, and artificial intelligence may help address these barriers and improve the reproducibility and practical utility of organoid-based evaluation systems.

Importantly, the near-term value of SkOs does not depend on achieving complete anatomical or functional equivalence to native human skin. The development of reconstructed human epidermis models has already shown that an *in vitro* model can still become useful in regulatory and industrial settings even if it does not reproduce the full complexity of skin biology. In a similar way, if SkOs can achieve acceptable batch-to-batch consistency through standardized engineering strategies, their human origin and appendageal complexity may offer advantages over traditional animal models in selected transdermal applications.

Nevertheless, progress toward broader implementation will require more than proof-of-concept studies. Multi-center validation, harmonized quality control criteria, and stronger correlations between organoid-derived data and human pharmacokinetic or clinical outcomes will all be necessary. At present, SkOs technology is still at an early translational stage, but its potential for more integrated and human-relevant evaluation deserves further investigation.

To support scalable production and higher-throughput use, future studies should move beyond a purely biological perspective and incorporate engineering, computational, and translational considerations. In particular, closer integration of stem cell developmental biology with microfluidic systems, digital twins, and artificial intelligence may improve model stability, physiological relevance, and quality control. Additional application-oriented studies are also needed to determine how SkOs perform in realistic transdermal evaluation settings. Such efforts may help move skin organoids from proof-of-concept systems toward more standardized and deployable evaluation platforms, thereby supporting the development and translation of novel transdermal formulations.
